# Chemical Constituents from the Stems of *Diospyros maritima*

**DOI:** 10.3390/molecules14125281

**Published:** 2009-12-15

**Authors:** Chi-I Chang, Chiy-Rong Chen, Hsi-Lin Chiu, Chao-Lin Kuo, Yueh-Hsiung Kuo

**Affiliations:** 1 Graduate Institute of Biotechnology, National Pingtung University of Science and Technology, Pingtung 912, Taiwan; E-Mail: changchii@mail.npust.edu.tw (C.-IC.); 2 Department of Biological Science and Technology, Meiho Institute of Technology, Pingtung 912, Taiwan; E-Mail: x2202@meiho.edu.tw (C.-R.C.); 3 Department of Chemistry, National Taiwan University, Taipei 106, Taiwan; 4 School of Chinese Medicine Resources, China Medical University, Taichung 404, Taiwan; E-Mail: clkuo@mail.cmu.edu.tw (C.-L.K.); 5 Tsuzuki Institute for Traditional Medicine, College of Pharmacy, China Medical University, Taichung 404, Taiwan; 6 Agricultural Biotechnology Research Center, Academia Sinica, Taipei 115, Taiwan

**Keywords:** *Diospyros maritima*, Ebenaceae, bis(6-hydroxy-2,3,4-trimethoxylphen-1-yl)methane, butylmethyl succinate

## Abstract

A new phenolic, bis(6-hydroxy-2,3,4-trimethoxylphen-1-yl)methane (**1**) and a new butanedioate, butylmethyl succinate (**2**), along with twenty-nine known compounds including one naphthoquinone derivative, two chromanones, eight benzenoids, one lignan, one tocopherol, and sixteen triterpenoids were isolated from the stems of *Diospyros maritima*. *epi*-Isoshinanolone (**3**) was isolated in pure form for the first time. In addition, 5,7-dihydroxy-2-methylchomanone (**4**) was isolated from a natural source for the first time. Their structures were established on the basis of spectroscopic data as well as direct comparison with authentic samples.

## 1. Introduction

*Diospyros maritima* Blume (Ebenaceae), a medium-sized shrub, is widely distributed throughout Southern Asia. The genus *Diospyros* is well-known to produce various naphthoquinone derivatives, some of which exhibit cytotoxic, ichthyotoxic, germination inhibitory, and antifungal activities [1,2,3,4]. The stems of *D. maritima* has been used in Taiwan as a folk medicine as a traditional treatment for rheumatic disease [5]. In previous studies, many naphthoquinones and triterpenes were isolated from its bark, root, fruits, leaves, and twigs by Tezuka [6] and Higa [1,2]. More recently, we also have reported some new naphthoquinones [7,8], triterpenes, steroids [3,9,10,11,12,13], phenolic and aliphatic components [14] from the stems of this plant and found that some naphthoquinones showed strong antitumor activity [3]. In continuation of our work on the discovery of secondary metabolites from this plant, we have also isolated a new phenolic, bis(6-hydroxy-2,3,4-trimethoxylphen-1-yl)methane (**1**), and a new butanedioate, butylmethyl succinate (**2**), along with twenty-nine known compounds including one naphthoquinone derivative, *epi*-isoshinanolone (**3**) [15], two chromanones, 5,7-dihydroxy-2-methylchromanone (**4**) [16] and 5-hydroxy-2-methylchromanone (**5**) [17], eight benzenoids, 1-(4,6-dihydroxy-2-methylphenyl)ethanone (**6**) [18], ethyl 2,4-dihydroxy-6-methyl-benzoate (**7**) [19], 4-hydroxybenzaldehyde (**8**) [20], vanillin (**9**) [21], 4-hydroxy-3,5-dimethoxy-benzaldehyde (**10**) [20], acetovanillone (**11**) [22], *trans*-coniferylaldehyde (**12**) [23], and (*E*)-3-(4-acetyloxy-3,5-dimethoxyphenyl)-2-propenal (**13**) [24], one lignan, 4-ketopinoresinol (**14**) [25], one tocopherol, α-tocopherol (**15**) [20], and sixteen triterpenoids: squalene (**16**) [20], lupeol caffeate (**17**) [26], betulin-28-acetate (**18**) [27], (*E*)-betulin-3β-*p*-coumarate (**19**) [28], (*Z*)-betulin-3β-*p*-coumarate (**20**) [28], betulinaldehyde (**21**) [29], 3-oxo-20(29)-lupen-28-oic acid (**22**) [20], betulinic acid (**23**) [30], betulic acid acetate (**24**) [31], 3-*O*-betulinic acid *p*-coumarate (**25**) [32], 3-*O*-palmitoylerythrodiol (**26**) [33], 28-*O*-acetylerythrodiol (**27**) [34], 3β-acetoxyolean-12-en-28-oic acid (**28**) [35], 3β-acetoxy-urs-12-en-28-oic acid (**29**) [36], 3β-hydroxyurs-12-en-28,13-olide (**30**) [37], and 3β-hydroxy-taraxastan-28, 20β-olide (**31**) [38]. 

## 2. Results and Discussion

The EtOH extracts of the stems of *D. maritima* was concentrated to give a black residue which was suspended in water and partitioned successively with *n*-hexane and *n*-BuOH. The combined *n*-BuOH soluble layer was subjected to repeated chromatography using silica gel and further purification by semipreparative HPLC to furnish two new compounds, bis(6-hydroxy-2,3,4-trimethoxylphen-1-yl)methane (**1**) and butylmethyl succinate (**2**), in addition to twenty-nine known compounds. The identification of the known compounds were performed by comparing their physical and spectral data (IR, UV, MS, and NMR) with literature values. This paper deals with the structural elucidation of compounds **1**-**4**. 

The HR-EI-MS of **1** exhibited a molecular ion peak at *m/z* 380.1476, which is corresponded to the molecular formula C_19_H_24_O_8_ and indicated eight degrees of unsaturation. The IR spectrum showed the presence of hydroxy (3,337 cm^-1^) and phenyl (1,619 and 1,500 cm^-1^) functionalities. The UV spectrum displayed the aromatic maximum absorption peak at 283 nm. In the ^1^H-NMR and DEPT spectra of **1**, the signals for five quaternary carbons (δ_C_ 110.4, 135.0, 149.5, 151.6, 152.8 ) and one tertiary carbon (δ_C_ 97.7) attributed a pentasubstituted benzene ring. The five substituents of the benzene ring included three methoxy groups (δ_H_ 3.75, 3.77, 4.07; δ_C_ 55.9, 61.1, 62.1), a hydroxyl (δ_H_ 8.10, disappeared on D_2_O exchange), and a methylene (δ_H_ 3.67; δ_C_ 18.3). From the above evidence, compound **1** was proposed to be a biphenyl derivative linked by a methylene group. The relative positions of those substituents on the benzene rings were determined by the long-range correlations between H-5 (δ_H_ 6.30) and C-1 (δ_C_ 110.4), C-3 (δ_C_ 135.0), and C-6 (δ_C_ 151.6); H-7 (δ_H_ 3.67) and C-1 (δ_C_ 110.4), C-2 (δ_C_ 149.5), and C-6 (δ_C_ 151.6) in the HMBC spectrum of **1**. Moreover, 2-OMe, 3-OMe, 4-OMe, and 6-OH also exhibited HMBC correlations with C-2, C-3, C-4, and C-6, respectively. The nOe correlations between H-7/6-OH (δ_H_ 8.10) and H-5/4-OMe (δ_H_ 3.77) further assured this proposed structure. Thus, compound **1** was elucidated as bis(6-hydroxy-2,3,4-trimethoxylphen-1-yl)methane.

The IR spectrum of **2** exhibited an absorption band (1735 cm^-1^) for an ester functionalty. The ^13^C-NMR and DEPT spectra indicated the presence of one methyl (δ_C_ 13.7), one methoxy (δ_C_ 51.8), five methylenes [δ_C_ 19.0, 28.9 (×2), 30.6, 64.6], and two quaternary carbons (δ_C_ 172.3, 172.8). A butoxyl group was elucidated by the COSY correlations between H-1΄ (δ_H_ 4.07) and H-2΄ (δ_H_ 1.57); H-2΄ and H-3΄ (δ_H_ 1.34); H-3΄ and H-4΄ (δ_H_ 0.88). From the above observations, compound **2** was considered as a butanedioate derivative with both methyl and butyl groups. The chemically equivalent signals of two methylene groups (δ_H_ 2.60, s) were assigned to be located between C-1 (δ_C_ 172.3) and C-4 (δ_C_ 172.8) according to their HMBC relationship. The proposed structure of **2** was also supported by the molecular ion peak at *m/z* 188 and the fragmental ion peak at *m/z* 157 [M-OMe]^+^, 129 [M-COOMe]^+^, 115 [M-OCH_2_ CH_2_ CH_2_ Me]^+^, 101 [M-CH_2_CH_2_COOMe]^+^, and 73 [M-COCH_2_CH_2_COOMe] ^+^ in the EI-MS spectrum. Compound **2** was accordingly identified as butylmethyl succinate.

The IR spectrum of **3** showed absorption bands at 3,360 (OH), 1,640 (conjugated ketone), and 1,580 (aromatic) cm^-1^. The ^1^H-NMR and ^1^H-^1^H COSY spectra displayed the signals for a set of aromatic ABX coupling system [δ_H_ 6.89 (1H, d, *J* = 7.4 Hz, H-7), 7.09 (1H, d, *J* = 7.4 Hz, H-5), 7.49 (1H, t, *J* = 7.4 Hz, H-6)] and a phenolic proton with strong intramolecular hydrogen bond [δ_H_ 12.35 (1H, s, 8-OH)]. In addition, the NMR signals for a complex coupling system attributing to a methine [δ_H_ 2.30 (1H, m, H-3)], which was coupled with methyl [δ_H_ 1.17 (3H, d, *J* = 6.6 Hz, 3-CH_3_)], oxymethine {δ_H_ 4.49 (1H, dd, *J* = 6.6, 6.9 Hz, H-4) coupled with 4-OH [δ_H_ 1.92 (1H, d, *J* = 6.9 Hz)]}, and methylene [δ_H_ 2.42 (1H, dd, *J* = 10.1, 17.3 Hz, H_a_-2); 2.90 (1H, dd, *J* = 4.0, 17.3 Hz, H_b_-2)] was observed. The above features were almost identical to those of the known compound, *epi*-isoshinanolone [15]. Thus, compound **3** was determined as *epi*-isoshinanolone. It was isolated in pure form for the first time. 

Analysis the IR spectrum of **4** suggested that it contains hydroxyl (3,400 cm^-1^), conjugated ketone (1,668 cm^-1^), and aromatic (1,620, 1,600 cm^-1^) functionalities. The ^1^H-NMR spectrum of **4** revealed the signals for an oxymethine [δ_H_ 4.66 (1H, m, H-2)] coupling to both methyl [δ_H_ 1.48 (3H, d, *J* = 5.7 Hz, 2-CH_3_)] and methylene [δ_H_ 2.84 (2H, m, H-3)], a phenolic proton with strong hydrogen-bonding [δ_H_ 11.19 (1H, s, 5-OH)] and a free phenolic proton [δ_H_ 5.90 (1H, s, 7-OH)], as well as two *meta*-positioned aromatic protons [δ_H_ 6.18 (1H, s, H-8); 6.29 (1H, s, H-6)]. The above spectral information was similar to that of 5,7-dihydroxy-2-methylchromanone previously reported in the literature [17]. Thus, compound **4** was characterized as 5,7-dihydroxy-2-methylchromanone. Compound **4** was isolated from the natural product for the first time in the present investigation. 

**Figure 1:**
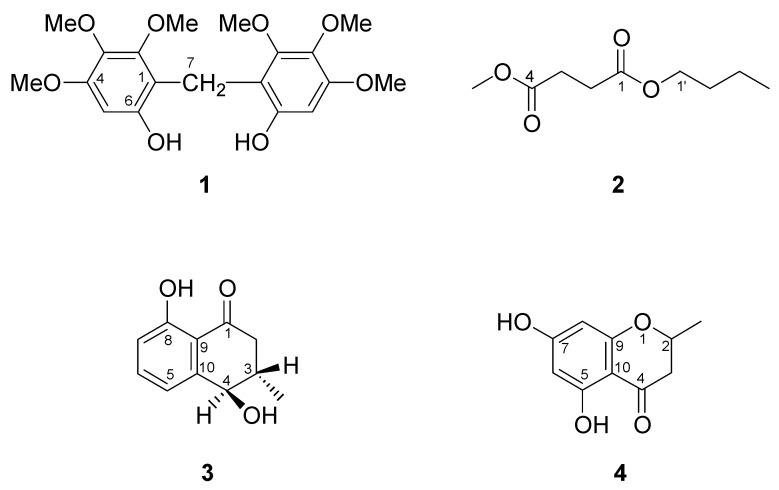
Chemical structures of compounds **1**-**4**.

## 3. Experimental

### 3.1. General

Melting points were determined with a Yanagimoto (MP500D) micromelting point apparatus and are uncorrected. IR spectra were recorded on a Perkin-Elmer 781 spectrophotometer. ^1^H- and ^13^C-NMR spectra were obtained in CDCl_3_ at a constant temperature controlled and adjusted to around 300 K on a Bruker AM-300 spectrometer, and the residual proton resonance (CHCl_3_) of CDCl_3_ was used as internal shift reference. The 2D NMR spectra were recorded on a Bruker DMX-300 spectrometer by using standard pulse sequences. EI-MS, FAB-MS, HR-EI-MS, UV spectra, and specific rotations were recorded on a JEOL JMS-HX 300, a JEOL JMS-HX 110, a JEOL SX-102A, a Hitachi S-3210 spectrometer, and a JASCO DIP-1000 digital polarimeter, respectively. TLC was performed by using Si gel 60 F_254_ plates (Merck). Column chromatography was performed on silica gel (Merck 9385, 70-230 mesh). HPLC was performed by using a Lichrosorb Si gel 60 (5μm) column (250 × 10 mm). 

### 3.2. Plant material

The stems of *D. maritima* were collected in Lin-Ko, Taiwan, in 1993. The plant material was identified by Muh-Tsuen Gun, formerly a technician of the Department of Botany, National Taiwan University, and a voucher specimen has been deposited at the National Research Institute of Chinese Medicine, Taipei, Taiwan, R.O.C.

### 3.3. Extraction and isolation

The stems of *D. maritima* (16 kg) were extracted three times with EtOH (160 L) at 60 °C for 10 h each time. The EtOH extract was evaporated under reduced pressure, yielding a black residue, which was suspended in H_2_O (12 L), and then partitioned (×5) with 1 L of *n*-hexane. The aqueous layer was partitioned (×4) again with 1 L of *n*-BuOH. The combined *n*-BuOH extracts (180 g) was then subjected to column chromatography over silica gel (120 × 6 cm) eluted with a setpwise gradient mixture of hexane and EtOAc as eluent. Seven fractions were collected as follows: 1 [3000 mL, hexane], 2 [4000 mL, hexane—EtOAc (9:1)], 3 [4000 mL, hexane—EtOAc (8:2)], 4 [4000 mL, hexane—EtOAc (7:3)], 5 [4000 mL, hexane—EtOAc (5:5)], 6 [3000 mL, hexane—EtOAc (3:7)], and 7 (6000 mL, EtOAc). Fraction 1 (8.5 g) was further purified through a silica gel column eluted with *n*-hexane/EtOAc (95/5) to yield **16** (6.2 mg). Fraction 3 (27.2 g) was further chromatographed on a silica gel column eluted with *n*-hexane/EtOAc (8/2) and semipreparative HPLC eluted with CH_2_Cl_2_/*n*-hexane/EtOAc (8/3/1) to obtain **8** (5.3 mg), **9** (3.0 mg), **10** (3.6 mg), **11** (5.1 mg), and **15** (4.2 mg). Fraction 4 (38.5 g) was subjected to column chromatography over silica gel eluted with *n*-hexane/EtOAc (7/3) and semipreparative HPLC eluted with CH_2_Cl_2_/*n*-hexane/EtOAc (3/3/1) to yield **3** (3.8 mg), **5** (3.5 mg), **6** (6.1 mg), **7** (5.0 mg), **12** (12.4 mg), **17** (3.0 mg), **18** (6.3 mg), **19** (4.0 mg), **20** (10.1 mg), **21** (4.7 mg), **22** (5.1 mg), **27** (4.1 mg), and **30** (3.0 mg). Fraction 5 (32.1 g) was further chromatographed on silica gel eluted with *n*-hexane/EtOAc (5/5) and semipreparative HPLC eluted with CH_2_Cl_2_/*n*-hexane/EtOAc (2/3/1) to yield **1** (5.2 mg), **2** (10.5 mg), **4** (3.2 mg), **13** (3.1 mg), **14** (6.2 mg), **23** (3.1 mg), **24** (3.2 mg), **25** (3.0 mg), **26** (3.5 mg), **28** (4.7 mg), **29** (2.8 mg), and **31** (4.0 mg). 

### 3.4. Spectroscopic data

*Bis(6-hydroxy-2,3,4-trimethoxylphen-1-yl)methane* (**1**): Gum; UV (logε) (MeOH) λ_max_: 283 (3.4) nm; IR ν_max_ cm^-1^: 3337, 1619, 1500, 1208, 1082, 897; ^1^H- and ^13^C-NMR (CDCl_3_): see Table 1; EI-MS (70 eV) *m/z* (rel. int. %): 380 [M]^+^ (38), 211 (23), 197 (39), 184 (100), 169 (42), 57 (25); HR-EI-MS *m/z* 380.1476 (calcd for C_19_H_24_O_8_, 380.1471).

*Butylmethyl succinate* (**2**): Colorless oil; IR ν_max_ cm^-1^: 1735, 1155, 1016; ^1^H-NMR (CDCl_3_): δ 0.88 (3H, t, *J* = 7.6 Hz, H-4’), 1.34 (2H, sex, *J* = 7.6 Hz, H-3’), 1.57 (2H, quin, *J* = 7.6 Hz, H-2’), 2.60 (4H, s, H-2, 3), 3.67 (3H, s, -OCH_3_), 4.07 (2H, t, *J* = 7.6 Hz, H-1’); ^13^C-NMR (CDCl_3_): δ 13.7 (C-4’), 19.0 (C-3’), 28.9 (C-2, 3), 30.6 (C-2’), 51.8 (-OCH_3_), 64.6 (C-1’), 172.3 (C-1), 172.8 (C-4); EI-MS (70 eV) *m/z* (rel. int. %): 188 [M]^+^(1), 157 (8), 129 (38), 115 (78), 101 (100), 87 (38), 73 (9), 55 (58); HR-EI-MS *m/z* 188.0940 (calcd for C_9_H_16_O_4_).

*epi-Isoshinanolone* (**3**): Amorphous solid; IR (dry film) ν_max_ cm^-1^: 3360, 1640, 1580; ^1^H-NMR (CDCl_3_): δ1.17 (3H, d, *J* = 6.6 Hz, 3-CH_3_), 1.92 (1H, d, *J* = 6.9 Hz, 4-OH), 2.30 (1H, m, H-3), 2.42 (1H, dd, *J* = 10.1, 17.3 Hz, H_a_-2), 2.90 (1H, dd, *J* = 4.0, 17.3 Hz, H_b_-2), 4.49 (1H, dd, *J* = 6.6, 6.9 Hz, H-4), 6.89 (1H, d, *J* = 7.4 Hz, H-7), 7.09 (1H, d, *J* = 7.4 Hz, H-5), 7.49 (1H, t, *J* = 7.4 Hz, H-6), 12.35 (1H, s, 8-OH); EI-MS (70 eV) *m/z* [M]^+^ 192 (90), 177 (20), 150 (45), 121 (100).

*5,7-Dihydroxy-2-methylchromanone* (**4**): Amorphous solid; IR (dry film) ν_max_ cm^-1^: 3400, 1668, 1620, 1600; ^1^H-NMR (CDCl_3_): δ1.48 (3H, d, *J* = 5.7 Hz, 2-CH_3_), 2.84 (2H, m, H-3), 4.66 (1H, m, H-2), 5.90 (1H, s, 7-OH), 6.18 (1H, s, H-8), 6.29 (1H, s, H-6), 11.19 (1H, s, 5-OH); EI-MS (70 eV) *m/z* 194 [M]^+^ (40), 185 (100), 149 (69).

## 4. Conclusions

Thirty-one compounds were isolated from the stems of *D. maritima*. Among them, bis(6-hydroxy-2,3,4-trimethoxylphen-1-yl)methane (**1**) and butylmethyl succinate (**2**) are new compounds and *epi*-isoshinanolone (**3**) was isolated in pure form for the first time. In addition, 5,7-dihydroxy-2-methylchromanone (**4**) was isolated from the natural source for the first time. This investigation of secondary metabolites may contribute to better understanding on the chemical characteristics of *D. maritima*.

## Figures and Tables

**Table 1:** ^1^H- and ^13^C-NMR spectral data for **1** (300 and 75 MHz in CDCl_3_).

position	δ_C_	δ_H_	position	δ_C_	δ_H_
1, 1’	110.4		7	18.3	3.67 s
2, 2’	149.5		2, 2’ -OCH_3_	62.1	4.07 s
3, 3’	135.0		3, 3’ -OCH_3_	61.1	3.75 s
4, 4’	152.8		4, 4’ -OCH_3_	55.9	3.77 s
5, 5’	97.7	6.30 s	6, 6’ -OH		8.10 s
6, 6’	151.6				
